# *Allium ekimianum*: a new species (Amaryllidaceae) from Turkey

**DOI:** 10.3897/phytokeys.62.7796

**Published:** 2016-04-06

**Authors:** Gülnur Ekşi, Mehmet Koyuncu, Ayşe Mine Gençler Özkan

**Affiliations:** 1 Department of Pharmaceutical Botany, Faculty of Pharmacy, Ankara University, Ankara, Turkey; 2 Faculty of Pharmacy, Cyprus International University, Haspolat-Lefkoşa, Cyprus

**Keywords:** Allium, section *Allium*, endemic species, taxonomy, Turkey

## Abstract

*Allium ekimianum* is described here as a new species. This taxon belongs to the genus Allium section Allium and grows in Elazığ Province (East Anatolia, Turkey). It is a narrowly distributed species and morphologically most similar to *Allium asperiflorum* and *Allium sintenisii*, and *Allium erzincanicum* but it is clearly differentiated due to the curved stem, smooth pedicel surfaces, bracteole arrangements at pedicel bases, tepal lengths and surfaces. In this study, a comprehensive description, distribution map of *Allium ekimianum*, identification key, and detailed illustrations are provided for *Allium ekimianum* and related taxa.

## Introduction

The genus *Allium* L. is one of the largest monocotyledonous genera with c. 900 species distributed world-wide (Govaerts et al. 2013, Keusgen et al. 2011). The genus was formerly included in the Liliaceae family, but the Angiosperm Phylogeny Group (APG) reassessed the taxonomic position of this genus and finally *Allium* was placed in the Amaryllidaceae family (APG III 2009). The primary evolution center of the genus extends across the Irano–Turanian biogeographical region, and the Mediterranean basin and western North America are secondary centres of diversity (Friesen et al. 2006). Based upon these centres, *Allium* species have scattered widely all over the northern hemisphere (Hanelt 1990, Fritsch and Friesen 2002). The genus is characterized by having bulbs enclosed in membranous (sometimes finally fibrous) tunics, terminal umbel, free or almost free, 1-veined tepals, often a subgynobasic style and loculicidal capsule with one or two seeds per loculus (Kollmann 1984).

Following the results of recent molecular investigations, *Allium* is divided into 15 subgenera and 56 sections (Friesen et al. 2006). Subgenus *Allium* is the largest, comprising approximately 280 species (Hanelt et al. 1992), 114 of which compose its largest section, *Allium* (Mathew, 1996). Section *Allium* encompasses those species of *Allium* that have a well-developed bulb, stem (never basal) leaves, campanulate to cup-shaped (never stellate) flowers, and filaments in two distinct whorls, the outer three nearly always simple and the inner three markedly tricuspidate (rarely 5-7 cuspidate) with the anther attached to the median cusp. This section includes economically important species, such as garlic (*Allium sativum* L.) and leek (*Allium ampeloprasum* L.), as well as other minor crops of local importance, such as great headed garlic, and kurrat (Block 2010). Despite the major importance of the section *Allium*, it has not been subjected to a comprehensive molecular taxonomic evaluation; only partial molecular genetic studies that involved a limited set of species have been published (Kik et al. 1997; Havey and Leite 1999; Bohanec et al. 2005; Hirschegger et al. 2010). Interspecific and infraspecific relationships within this section still remain unresolved. As reviewed by Mathew (1996), polyploidy is a common feature in section *Allium*. However, to a certain extent, it has been left unexplored, leaving the origin of polyploid species undetermined (Hirschegger et al. 2010).

Turkey has approximately 190 *Allium* taxa in 14 sections, c. one-third endemic, demonstrating that it is a prominent part of the southeastern Asian center of *Allium* diversity (Ekşi et al. 2015; Özhatay and Kandemir 2015; Koyuncu 2012). Section *Allium* remains the most species-rich section of the genus (Friesen et al. 2006).

Turkey has four reasons for having an exceptionally rich flora. First, it is the meeting point of three phytogeographical regions, the Euro–Siberian, Mediterranean and Irano–Turanian regions. Second, Anatolia (Asian part of Turkey) is a passageway and a migration route between Southern Europe and the flora of South–West Asia allowing the penetration of Asiatic elements into South Europe. Third, many taxa have their center of origin and/or center of diversity in Anatolia. Fourth, the endemism ratio is high, presumably connected with the climatic and topographical diversity of the country (Davis 1965, 1971).

During the revision of the treatment of *Allium* in Turkey, individuals of a new species were collected by Prof. Dr. Mehmet Koyuncu in 1983 from Eastern Anatolia. They belong to Allium section Allium due to ovoid bulb, linear leaves, campanulate to ovoid perigon; 3–cuspidate inner flaments, distinct nectariferous pores on ovary, ovule numbers in per loculus (Kollmann 1984). The initial evaluation suggested this collection was a form of *Allium asperiflorum* Miscz. However, detailed examination of herbarium material and a review of the literature indicated this represented an undescribed species. The present study is focused on the morphological characters for distinguishing a new species in Allium section Allium. Investigations on living and herbarium specimens suggest that this new species is morphologically most similar to *Allium asperiflorum*, *Allium erzincanicum* N. Özhatay & Kandemir and *Allium sintenisii* Freyn.

## Materials and methods

**Figure 1.**
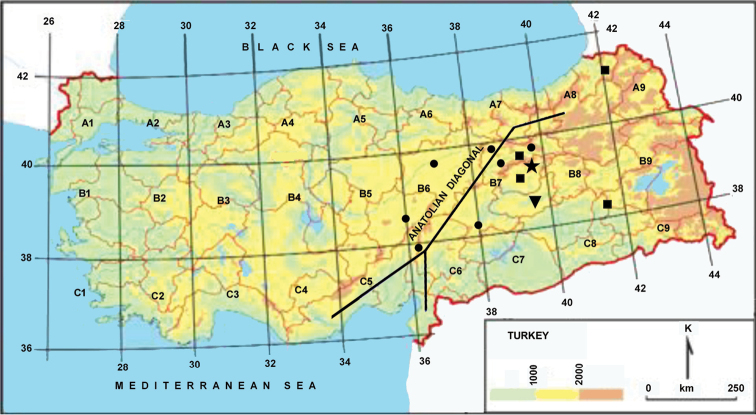
Distribution map of *Allium ekimianum*, *Allium asperiflorum*, *Allium sintenisii*, *Allium erzincanicum*.

The overall morphology of the new species was examined by stereo binocular microscope (Leica Zoom 2000). For morphological comparisons, we consulted dry herbarium material kept in AEF, ANK, E, GAZI, and ISTE (acronyms according to Thiers 2015). The *Flora of Turkey* and floras of the neighbouring regions including Iran, Iraq, and Syria were also consulted (Boissier 1882, Feinbrun 1948, Wendelbo 1971, 1985, Kollmann et al. 1983, Kollmann 1984, Mathew 1996, Özhatay and Tzanoudakis 2000). The diagnostic traits of the new species and its three most similar relatives are presented in Table 1. Distribution map of compared taxa is provided according to the Grid classification system developed by Davis (1965) in Figure 1. IUCN conservation assessment and justification is proposed according to the criteria established by IUCN (IUCN 2003). In addition, an identification key is provided to distinguish new species and closely related taxa.

**Table 1.** Main differences between *Allium asperiflorum*, *Allium ekimianum* and *Allium sintenisii*, *Allium erzincanicum*.

	*Allium asperiflorum*	*Allium ekimianum*	*Allium sintenisii*	*Allium erzincanicum*
Outer bulb tunics	membranous	membranous	membranous	reticulate-fibrous
Stem	erect	curved	almost erect	erect
Leaves	densely scabrid	glabrous	densely scabrid	glabrous or almost glabrous
Bracteoles	solitary at the base of each pedicel	outer ones with united bracteoles at base	outer ones with united bracteoles at base	outer ones with united bracteoles at base
Pedicel surface	papillose	smooth	scabrid near the base of perianth	smooth
Outer tepal	densely papillose, keeled	verrucose–scabrid, straight	loosely bearded with long white papillae at whole surface, keeled	loosely bearded with long white papillae just on the midvein, keeled
Inner tepal	papillose, 5–7 × 2.5 mm, equal to outer tepal	smooth, c. 4 × 2 mm, distictly longer than outer tepal	smooth, 7–10 × 3 mm, almost equal to outer tepal	loosely bearded with long white papillae just on the midvein, 4–5 × 1–2 mm, equal to outer tepal

## Taxonomic treatment

### Allium ekimianum

Taxon classificationPlantaeAsparagalesAmaryllidaceae

Ekşi, Koyuncu & Özkan sp. nov.

urn:lsid:ipni.org:names:60471703-2

Figure 2

#### Note.

Diagnostic characters for *Allium ekimianum* include curved stem, smooth pedicels, united bracteoles, verrucose–scabrid and straight outer tepal, smooth inner tepal, longer inner tepal.

#### Type.

Turkey. Elazığ: Fırat University, steppe, c. 1150 m, 02.07.1983, *Koyuncu 7847* (holotype: AEF!, isotype: GAZI!).

#### Description.

Bulb ovoid, 0.7–1.2 × 1-1.5 cm; outer tunics membranous, brownish, ± breaking into parallel fibres; inner tunics white; bulblets absent. Stem 15–35 cm, curved, often purplish below. Leaves 2–3, linear, 1–2 mm broad, flat, shorter than scape, sheathing lower ½ of stem. Umbel globose–subglobose, 1.5–3 cm diameter, dense, 20-60 flowered. Spathe caducous. Pedicels smooth, unequal, not elongating in fruit; up to 2.5 × perigon; bracteoles present, united at the base of outer pedicels, splitting into several lobes at apex, c. 5 mm. Perigon ovoid, campanulate; tepals purple, pale pink; outer tepals straigth, 5 × c. 3.5 mm, obovoid, verrucose–scabrid, acute–subacute, obtus at apex; inner tepals c. 4 × 2 mm, narrowly oblong, smooth, obtus at apex. Stamens included; filaments ciliate at base; inner flaments 4 × 2 mm; median cusps c. 1 mm, slightly shorter than lateral cusps (c. 1.5 mm); basal lamina c. 3 mm, 3 times longer than median cusps. Anther 1 mm, yellow. Pistil c. 3–5 mm; style c. 1–2 mm; ovary c. 2–3 × 1.5–2 mm, ovoid, smooth. Capsule 4 × 3.5 mm, ovoid; valves emarginate–bilobate at apex; seed 3 × 1.5 mm, black.

#### Etymology.

The species is named in honor of the eminent Turkish botanist Prof. Dr. Tuna Ekim, who dedicated his life to Turkish Flora, was retired from İstanbul University.

#### Distribution and ecology.

The distribution of *Allium ekimianum* is restricted to Province of Elazığ from East Anatolia, where it grows on steppe between 1100–1200 m of elevation. Species associated with *Allium ekimianum* include *Campanula stricta* L., *Silene italica* (L.) Pers., *Silene vulgaris* (Moench) Garcker, *Euphorbia macroclada* Boiss., *Papaver rhoeas* L., *Crataegus monogyna* Jaq., *Rosa canina* L., Rosa × dumalis Bechst., *Potentilla erecta* L., *Sanguisorba minor* Scop., *Achillea millefolium* L., *Allium scorodoprasum* L., *Vicia cracca* L., *Crepis foetida* L., *Eryngium campestre* L., *Salvia verticillata* L., *Avena sterilis* L. Elazığ is located on the east of Anatolian diagonal, in the skirts of South-Eastern Taurus Mountains (Çakılcıoğlu et al. 2008), in the Upper Euphrates Region of the Eastern Anatolia Region (Şengün 2007). Elazığ belongs to the Irano-Turanian Plant Geography Region and falls within the B7 grid square (Davis 1965). The Irano-Turanian Region is confined to Central and East Anatolia. This great region of steppe, mountain steppe and semi-desert is also characterized by the existence of a hypothetical oblique line that runs from Bayburt-Gümüşhane southwestwardly to Anti-Taurus where it bifurcates with one prong leading to the Amanus and the other to the Cilician Taurus. This line is called “Anatolian Diagonal” (Figure 1). The flora of central Anatolia as the western side of the Diagonal is floristically different from the rest of the Irano-Turanian region to the east. According to the plant distribution patterns in eastern Anatolia, many endemics are restricted to part of the Diagonal belt, or extend right along it (Davis 1965, Davis 1971).

#### IUCN Conservation Assessment and Justification.

Following the criteria established by IUCN (IUCN 2003), an initial provisional assessment of Critically Endangered (CR) (criteria B2a + B2biii) is suggested for this new taxon. This species occurs only in Elazığ University campus area in Elazığ province (East Anatolia) at 1100–1200 m. The area is under subversive people activities such as new constructions of buildings. As a result, the habitat of *Allium ekimianum* is highly threatened of vanishing by people activities. The area of *Allium ekimianum* occupancy (AOO) is less than 10 km^2^ with the number of mature individuals which is under reduction and being less than 50.

#### Related species.

**Figure 2.**
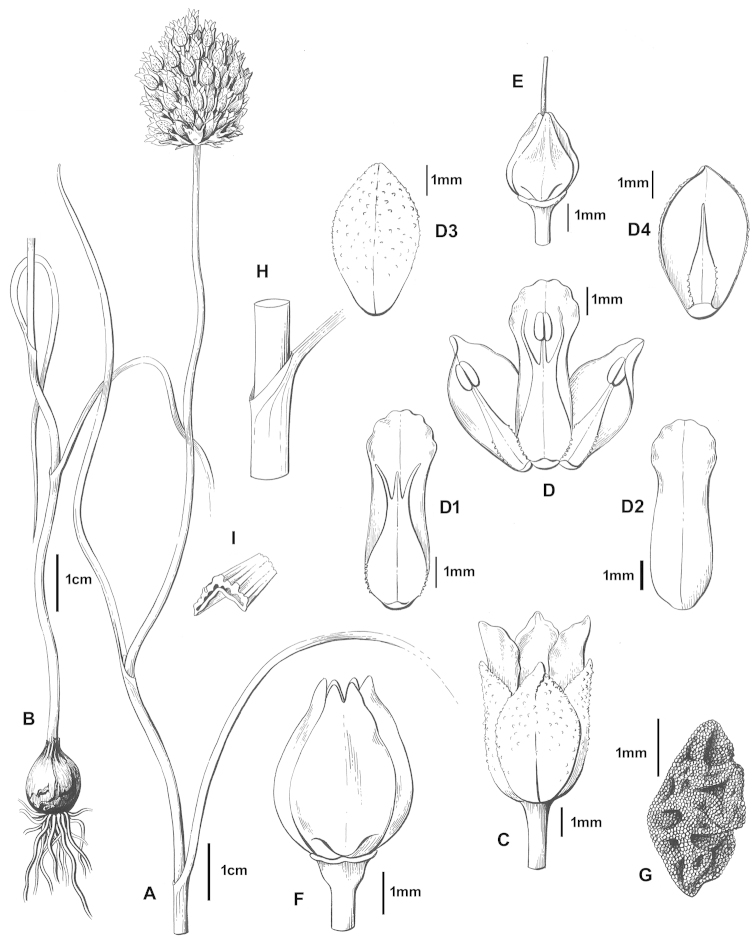
*Allium ekimianum* (*Koyuncu 7847*/Elazığ). Plant (**A, B**), flower (**C**), flower longitudinal section (**D**), inner tepal (**D1, D2**), outer tepal (**D3, D4**), pistil(**E**), capsule (**F**), seed (**G**), leaf sheathing (**H**), leaf cross section (**I**). (Drawn by Gülnur Ekşi).

**Figure 3.**
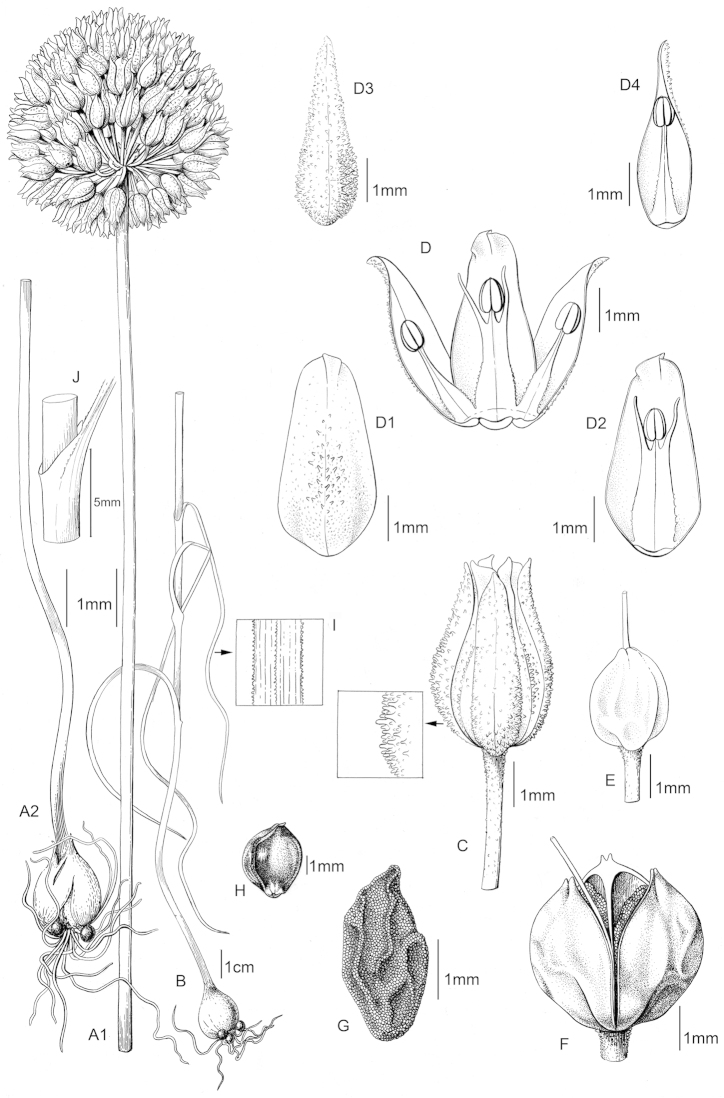
*Allium asperiflorum* (*Koyuncu 10539*/Artvin). Plant (**A1, A2, B**), flower (**C**), flower longitudinal section (**D**), inner tepal (**D1, D2**), outer tepal (**D3, D4**), pistil (**E**), capsule (**F**), seed (**G**), bulblet(**H**), leaf surface (**I**), leaf sheathing (**J**). (Drawn by Gülnur Ekşi).

**Figure 4.**
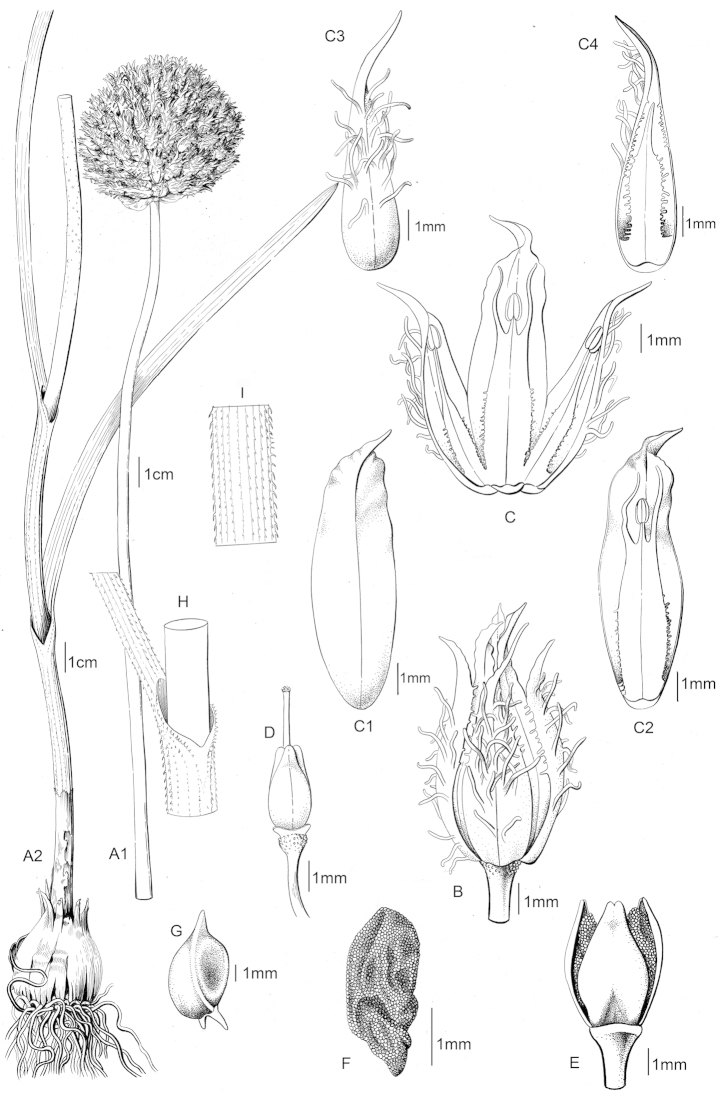
*Allium sintenisii* (*Koyuncu 9692*/Kahramanmaraş). Plant (**A1, A2**), flower (**B**), flower longitudinal section (**C**), inner tepal (**C1, C2**), outer tepal (**C3, C4**), pistil (**D**), capsule (**E**), seed (**F**), bulblet (**G**), leaf sheathing (**H**), leaf surface (**I**). (Drawn by Gülnur Ekşi).

*Allium ekimianum* is closely related to *Allium asperiflorum* and *Allium sintenisii* and *Allium erzincanicum*. All four species share traits of ovoid bulb, globose to subglobose umbel, campanulate to ovoid perigon, rough outer tepal surfaces, stamens sorter than perigon, ovoid ovary. *Allium ekimianum* differs from *Allium asperiflorum*, *Allium sintenisii* and *Allium erzincanicum* in its outer tunics, stem, leaves, bracteoles, pedicel surface, outer tepal, and inner tepal characters. The three species are compared in Table 1 and these traits are illustrated in Figures 2–4.

### Key for identification of *Allium* species related to *Allium ekimianum*

**Table:** 

1	Outer perianth segments loosely bearded at whole surface or along midvein with long white papillae, pale pink, more intensely pink at tip	**2**
–	Outer perianth segments not bearded on whole surface or along midvein	**3**
2	Perigon 7–10 mm; outer tunics membranous; leaves scabrid; pedicels scabrid near the base of perianth; outer tepal loosely bearded with long white papillae at whole surface; inner tepal smooth	***Allium sintenisii***
–	Perigon 4–5 mm; outer tunics reticulate fibrose; leaves glabrous or almost glabrous; pedicels smooth; outer tepal loosely bearded with long white papillae just on the midvein; inner tepal bearded on the midvein	***Allium erzincanicum***
3	Stem erect; involucre–like structure absent; outer tepal keeled, surface densely papillose; inner tepal surface scarcely papillose; leaves densely scabrid; pedicels densely papillose; bulblets numerous	***Allium asperiflorum***
–	Stem curved; involucre–like structure present; outer tepal not keeled, surface verrucose–scabrid; inner tepal surface glabrous; leaves glabrous; pedicels smooth; bulblets ± present	***Allium ekimianum***

### Additional specimens examined

The capital letters and the numbers in bold after species names represent the Grid classification system (Davis 1965) and the names in bold are the provinces from the Eastern Turkey. The abbreviations and the numbers in brackets at the end of the sentences represent the herbarium names and the accession numbers, respectively.

***Allium asperiflorum*: A9 Artvin**: Borçka–Artvin arası, Artvin’e 10 km kala, kayalıklar, 200 m, 14 vii 1993, *M. Koyuncu 10539* (AEF 18113). **B6 Sivas**: Divriği–Cürekarası, 3 vi 1983, *H. Başer s.n.* (ESSE 3320). **B7 Erzincan**: İliç, Hassanova village, 900–1100 m, *Çelik s.n.* (AEF 5699). İliç, Hasanova Köyü altındaki Tepeler, 900–1000, 16 vi 1976, *N. Çelik s.n.* (AEF 5699). Erzincan–Refahiye yolu, 35 km, kuruçakıllı yamaçlar, 1900 m, 22 viii 1990, *M. Koyuncu 8808* (AEF 15737). İliç–Refahiye çevresi, Gümüşahar’dan sonar Sunibeli Geçiti, orman açıklıkları, 1700 m, 21 vi 2005, *M. Koyuncu* 15098 & *N. Aslan* (AEF 24263). **B7 Tunceli**: Ovacık üzeri, Munzur Dağı, Kepir Gediği, kayalık taşlık arazi, 2400–2750 m, 10 viii 1976, *M. Koyuncu & N. Çelik s.n.* (AEF 5683). **C8 Siirt**: Pervari’nin üstü, kalker kayalıklar, stepler, 1600–1700 m, 16 vi 1980, *M. Koyuncu 3260* (AEF 9563). ***Allium sintenisii*: B6 Kayseri**: Sarız, Yalak, Binboğa Dağı, 2000–2200 m, 1 vii 1992, *M. Koyuncu & H. Duman 5175* (AEF 17830). Bakır Dağı–Tufanbeyli arası, Gezbeli Geçidi, 2200 m, 28 vii 2008, *M. Koyuncu 15993* (AEF 25277). **B6 Malatya**: Kuluncak, Kızılyüce Dağı kuzey eteği, çayırlık, 1700 m, 18 vi 1994, *B. Yıldız 11582* (AEF 26253). **B6 Maraş**: Göksun–Binboğa Dağı, 2000–2400 m, 17 vii 1992, *M. Koyuncu 9692, H. Duman, Z. Aytaç* (AEF 17465). **B7 Malatya/Sivas**: Kangal to Hekimhan, 1300 m, *Stn. & Hend. 5390.* **B7 Erzincan**: Spikör Dağı Geçidi, step yamaçlar, 2300 m, 27 viii 2008, *M. Koyuncu 16128* (AEF 25453) **ibid.** 14 vii 2009, *M. Koyuncu 16176* (AEF 25593). ***Allium erzincanicum*: B7 Erzincan**: Munzur Dağları, Mercan Suyu, kalker kayalıklar, 37 S 550540 D, 4374863 K, 1997 m, 08 vii 2014, *Kandemir 10613* (isotype NGBB).

## Supplementary Material

XML Treatment for Allium ekimianum
